# Flexural Behavior of 3D-Printed Carbon Fiber-Reinforced Nylon Lattice Beams

**DOI:** 10.3390/polym16212991

**Published:** 2024-10-25

**Authors:** Muhammet Muaz Yalçın

**Affiliations:** Department of Mechanical Engineering, Sakarya University, 54050 Serdivan, Turkey; myalcin@sakarya.edu.tr; Tel.: +90-264-295-5858

**Keywords:** 3-point bending, cubic lattice topology, octet lattice topology, energy absorption performance, chopped carbon fiber reinforced

## Abstract

This study investigates the flexural behavior of 3D-printed multi-topology lattice beams, with a specific emphasis on octet and cube lattice geometries created through fused deposition modeling (FDM). The mechanical properties of these beams were evaluated through quasi-static three-point bending tests. A comparative analysis of load-carrying capacity, energy absorption, and specific energy absorption (SEA) indicates that octet lattice beams exhibit superior performance to cube lattice beams. The octet lattice beam in the triple-layer double-column (TL-DC) arrangement absorbed 14.99 J of energy, representing a 38% increase compared to the 10.86 J absorbed by the cube lattice beam in the same design. The specific energy absorption (SEA) of the octet beam was measured at 0.39 J/g, which exceeds the 0.29 J/g recorded for the cube beam. Two distinct types of deformations were identified for the struts and the beam layers. Octet struts exhibit enhanced performance in stretch-dominated zones, whereas the cube system demonstrates superior efficacy in compressive-dominated regions. The results highlight the enhanced efficacy of octet lattice structures in energy absorption and mechanical stability maintenance. The investigation of sandwich lattice topologies integrating octet and cube structures indicates that while hybrid designs may exhibit efficiency, uniform octet structures yield superior performance. This study provides valuable insights into the structural design and optimization of lattice systems for applications requiring high-energy absorption and mechanical robustness.

## 1. Introduction

The advent of 3D printing technology has revolutionized the manufacturing of structural components, particularly beams, which are critical in various engineering applications. This technology allows for the creation of complex geometries and tailored mechanical properties that are difficult to achieve with traditional manufacturing methods. As a result, 3D-printed beams have garnered significant attention, especially in terms of their bending performance, which is crucial for structural integrity and reliability in real-world applications.

Material selection is one of the most critical factors influencing the bending behavior of 3D-printed beams. Various studies have explored the mechanical properties of beams fabricated from different materials, including thermoplastics, composites, and concrete [1,2,3,4,5,6,7,8,9,10,11]. For instance, Wu et al. [4] investigated the bending properties of fiber-reinforced resin T-beams and found that the incorporation of continuous fibers significantly enhanced mechanical performance compared to traditional materials. Similarly, Zhang demonstrated that variable cross-section I-beams reinforced with continuous and short fibers exhibited improved load-bearing capabilities [12]. The use of natural fibers in composite face sheets, as studied by Kamarian, further highlighted the potential for lightweight and strong structural components in sandwich beams [13].

The geometry of the beam is another crucial determinant of its bending performance. Research has shown that varying the cross-sectional shape of beams, such as using I-beams or T-beams, can optimize their load-bearing capabilities while minimizing material usage [2,4,6,14]. Najafi et al. demonstrated that the use of auxetic cores in sandwich beams enhances energy absorption and stiffness, underscoring the importance of innovative design approaches in maximizing the mechanical properties of 3D-printed beams [6]. Additionally, complex geometries like lattice structures and honeycomb patterns have been shown to improve the bending characteristics of 3D-printed beams, as evidenced by the work of Egan et al. [15]. The printing process itself introduces additional variables that can significantly impact the mechanical properties of 3D-printed beams. Factors such as layer height, infill density, and printing speed play a critical role in determining the structural performance of the final product [15,16,17,18,19,20]. Liu and Jiang [21] found that adjusting the infill density and layer thickness influenced the tensile and bending strength of polylactic acid (PLA) beams. Ergene et al. [22] highlighted the importance of optimizing printing parameters, such as print orientation, to achieve desired mechanical properties in PET-G tapered beams. These findings underscore the need for the precise control of printing conditions to ensure the consistent and reliable performance of 3D-printed components. Reinforcement strategies are another essential aspect of enhancing the bending capabilities of 3D-printed beams [19,23,24,25,26]. Additionally, the use of continuous carbon fiber-reinforced polymers (CFRPs) has been demonstrated to enhance the flexural properties of beams, making them more suitable for load-bearing applications [14,16,17].

The dynamic behavior of 3D-printed beams under bending loads is another vital area of research. Liu and Jiang [21] investigated the thermoplastic composite beams and revealed that incorporating magnetorheological elastomer cores could allow for controlled bending responses under varying external conditions. This innovative approach opens new avenues for developing adaptive structures capable of responding to environmental stimuli, enhancing their functionality in real-world applications. The use of 3D-printed auxetic materials in beam design also offers potential benefits for improving energy absorption and dynamic performance [27,28,29,30,31,32]. The application of machine learning techniques to predict the bending behavior of 3D-printed beams is an emerging area of research [13,33]. Kamarian utilized machine learning algorithms to model the bending characteristics of sandwich beams, demonstrating the potential for data-driven design approaches that optimize material usage and structural performance [13]. This represents a significant advancement in the design of high-performance structural components by combining advanced computational methods with traditional experimental approaches [33,34]. The predictive capabilities of machine learning could also help identify optimal design parameters for specific applications, reducing the need for extensive physical testing [35].

Environmental sustainability is another crucial consideration in the development of 3D-printed beams. With the increasing demand for eco-friendly construction practices, researchers are exploring the use of bio-based and recycled materials in 3D printing. For example, Liu et al. emphasized the importance of using sustainable materials without compromising mechanical performance [36]. The use of recycled polymers and natural fibers in 3D printing aligns with global efforts to reduce the carbon footprint of construction practices, making this an essential area of ongoing research [37,38]. The potential for using biodegradable materials in 3D-printed beams also offers opportunities for creating more sustainable and environmentally friendly structures [39]. In addition to material sustainability, the durability and long-term performance of 3D-printed beams are critical areas of study. The aging and degradation of materials over time can significantly impact the mechanical properties of beams, particularly in harsh environmental conditions [40]. Research has shown that exposure to factors such as UV radiation, moisture, and temperature fluctuations can lead to a reduction in bending strength and overall durability [20]. Understanding these factors is crucial for designing 3D-printed structures that maintain their performance over extended periods.

In conclusion, the bending behavior of 3D-printed beams is influenced by various factors, including material selection, geometric design, printing parameters, reinforcement strategies, and environmental considerations. Ongoing research in these areas continues to expand our understanding of how to optimize the mechanical properties of 3D-printed beams, paving the way for their broader application in structural engineering. As technology advances, the integration of machine learning and sustainable materials will likely play a pivotal role in the future development of 3D-printed structural components.

## 2. Materials and Methods

### 2.1. Lattice Parent Material

**Figure 1:**
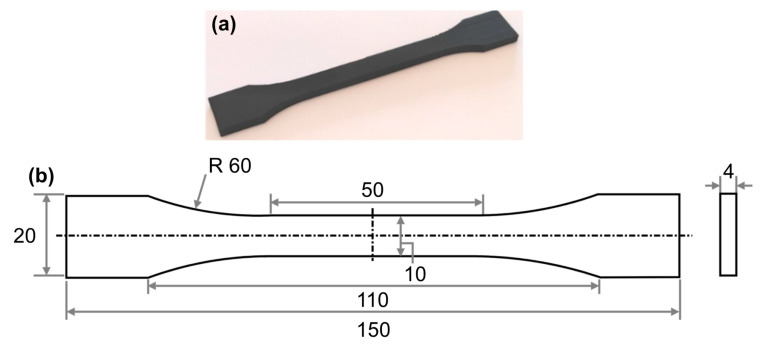
(**a**) Tensile specimen printed with Onyx filament and (**b**) ISO 527-2 Type B standard tensile test specimen dimensions in [mm].

The lattice parent material was a nylon-based composite carbon fiber filament Onyx™ from Markforged, Ottawa, ON, Canada. The length of the carbon fibers in Onyx varies from 8 to 45 µm, and fibers are mainly oriented in the deposition direction of the material. The carbon fiber whiskers enhance the strength and durability of the nylon. The Onyx material allows for good accuracy and surface finish of the produced parts. Dog-bone tensile specimens were fabricated with the flat orientation per the specifications of ISO 527-2 [23], illustrated in Figure 1, to obtain the mechanical properties of Onyx material.

The specimens were tensile tested at a rate of 2 mm/min on an Instron machine (model number 3367) with a capacity of 30 kN. Tests were conducted until failure, and tests were repeated three times to ensure the accuracy of results. The load-displacement data were collected directly from the Instron machine. A video extensometer was also used to obtain the strain data and calculate the Poisson’s ratio. The general mechanical properties of the Onyx material are summarized in Table 1.

### 2.2. Printing Lattice Specimens

**Figure 2:**
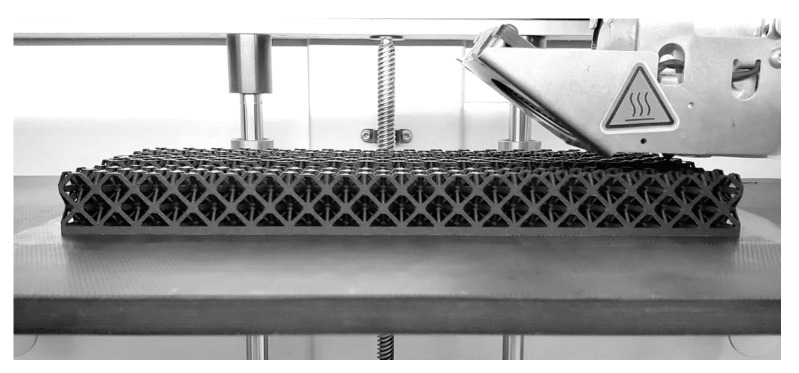
Three-dimensional printing process of multi-layer octet lattice beam (O3).

A 3D printer model, Onyx One™ from Markforged, was used for the production of the lattice beams. Both the tensile test specimens and the lattice beam specimens were produced using the Fused Deposition Modeling (FDM) technique. The manufacturing process of the octet lattice beam can be seen in the figure below (Figure 2). As can be seen from the figure, three specimens were produced at one time to eliminate possible defects based on the production method and conditions so that all specimens were identical. In addition, 3D printing parameters in the production process of the beam specimens are detailed in Table 2.

**Figure 3:**
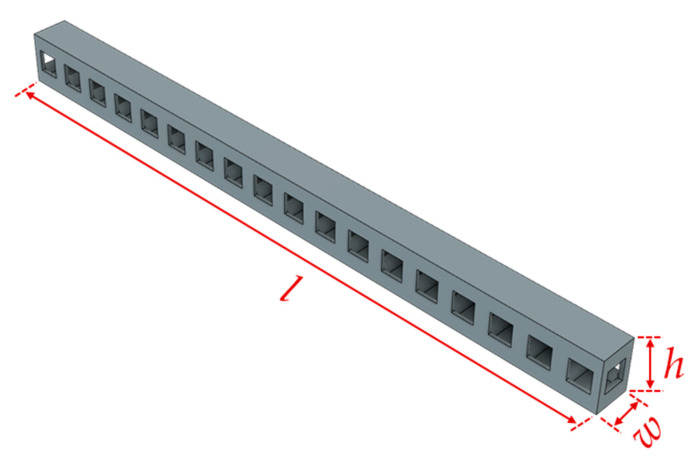
Schematic view of monolayer single-column cube beam.

The beams to be used in the 3-point bending test were produced in two different lattice structures, cube and octet, and three different specimen combinations were determined for each type of lattice. The cube and octet lattices had a length of the unit cell of 10 mm. All the specimens used in the experimental study were produced at a relative density of 30%, taking into account the production capability of the printer, so their weights were almost equal to each other, although there were very small deviations. The lattice beams were printed together with layers with a thickness of 2 mm on the upper and lower surfaces of the beams. The cube and octet lattices had a strut diameter of 2.05 mm and 0.87 mm, respectively [16]. The reason for this difference was that these lattices had different numbers of struts while they had the same relative density. There were three different groups of beams according to the unit lattice numbers in width (*w*) and height (*h*) directions, and all of them had 18 units of lattice in the length (*l*) direction. The first group of beam specimens (monolayer single column) had one lattice in the height and width directions (Figure 3). The second group (monolayer double column) and the third group (triple-layer double column) groups of beam specimens had two-unit lattices in the width direction. The second group of beams had a unit lattice in the height direction, while the third group had a three-unit lattice. The dimensions of all lattices are given together in Table 3.

**Figure 4:**
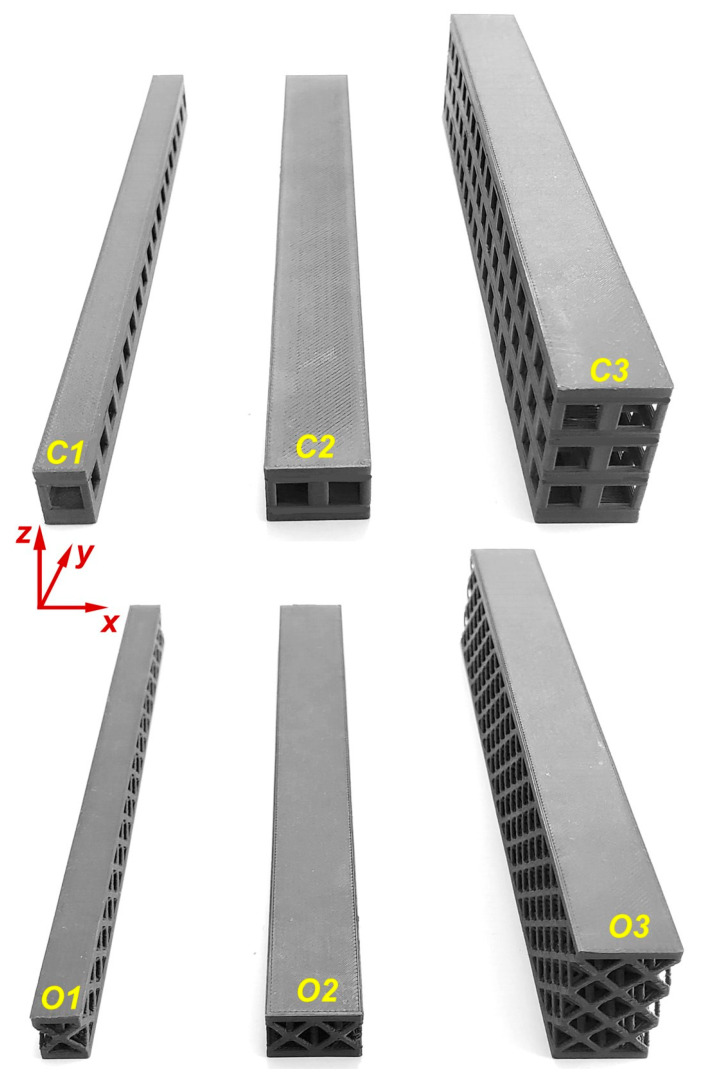
Pre-experiment images of the monolayer single column, monolayer double column, and triple-layer double-column cube (first line) and octet (second line) lattice beams.

**Figure 5:**
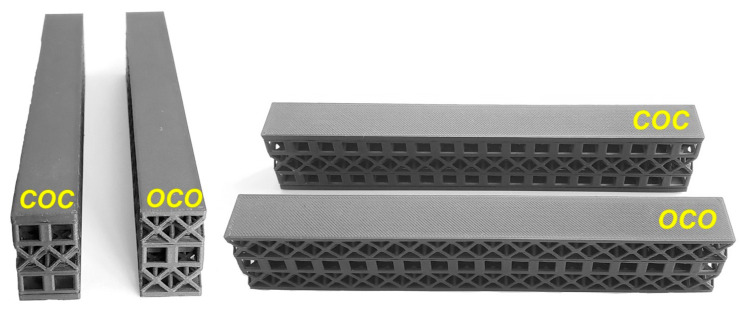
Sandwich lattice beam structures (COC and OCO).

In order to see the effect of different lattice types in more detail, sandwich model beams using cube and octet lattices together were also produced in the third group. All the specimens produced with the 3D printer can be seen together in Figure 4 (single- and triple-layer cube and octet lattices) and Figure 5 (sandwich lattice beams of cube-octet-cube (COC) and octet-cube-octet (OCO)) below.

### 2.3. Experimental Testing Set-Up

**Figure 6:**
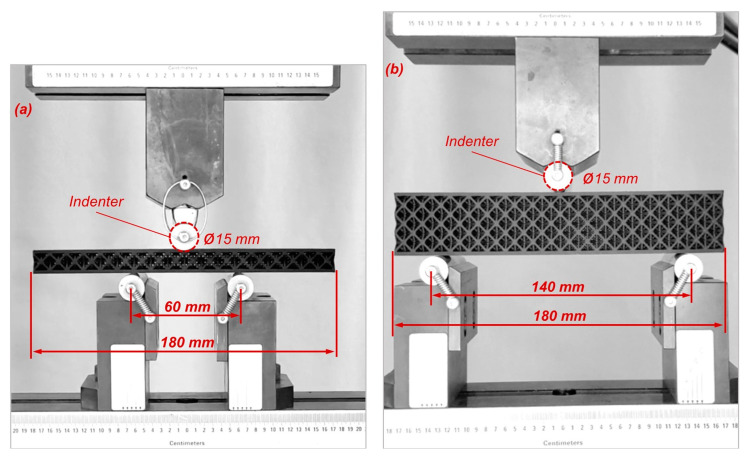
The overall view of the three-point bending test setup: (**a**) monolayer beams, (**b**) triple-layer beam.

The three-point bending tests were performed using an MTS testing machine (Istanbul, Turkey), following the related ASTM D7249/C0393 standards [41,42]. Due to the printing capability of the printer that was used in this study (Markforged Onyx One™ printer (building area 154 × 132 × 320 mm)), beam specimens were scaled down as performed in similar studies [27,43]. The ratio of the space between the fixed supports to the height of the beams was set to 4 as performed in similar studies [6,13,28,43,44,45]. In the present study, two cylindrical-shaped supports with a radius of 15 mm were fixed at 60 mm and 140 mm for the monolayer (C1, C2 and O1, O2) and triple-layer (C3, O3, and sandwich beams) beams, respectively. A cylindrical indenter with a diameter of 15 mm applied quasi-static loading on the specimens at a specific displacement rate of 2 mm/min. Since there was a 60 mm space between the fixed supports in the 3-point bending test of monolayer beams, the indenter can only move downward in a limited displacement range. An apparatus was placed between the indenter and the support that the indenter was mounted onto it to solve this issue (Figure 6a). Since the lattice specimens were not conducive to the direction collection of strain data by extensometer, the force–displacement data were collected directly from the measurements recorded by the MTS machine. The overall view of the three-point bending test setup (with a monolayer octet lattice) is shown in Figure 6.

### 2.4. Assessment of Crashworthiness Criteria

Finding the crashworthiness indication is essential in examining a structure’s crashworthiness. Three parameters—load-carrying capacity (LC), energy absorption (EA), and specific energy absorption (SEA)—were proposed to qualitatively examine the crashworthiness of the beam constructions under three-point bending performance, as the literature [27,28,29] indicates. Three distinct indications were employed in this study to evaluate auxetic beam crashworthiness. The highest force that occurs through the force story of the structure is known as its load-bearing capacity or LC. The EA indicates the energy absorbed by the lattice beam structure for a specific displacement value, considering the force–displacement curve. Hence, the *EA* can be explained as follows:

(1) $$EA=\int_{0}^{d}F\left(y\right)\;dy$$

where *F*(*y*) is the instantaneous load carried by the beam structure and *d* is the compression displacement. The specific energy absorption, described as the energy absorbed per unit mass, has been broadly used as follows:

(2) $$SEA=\frac{EA}{m}$$

where *m* is the mass of the lattice beam structure and is calculated for the length between the fixed supports.

## 3. Results and Discussion

### 3.1. Deformation of the Lattice Beams

**Figure 7:**
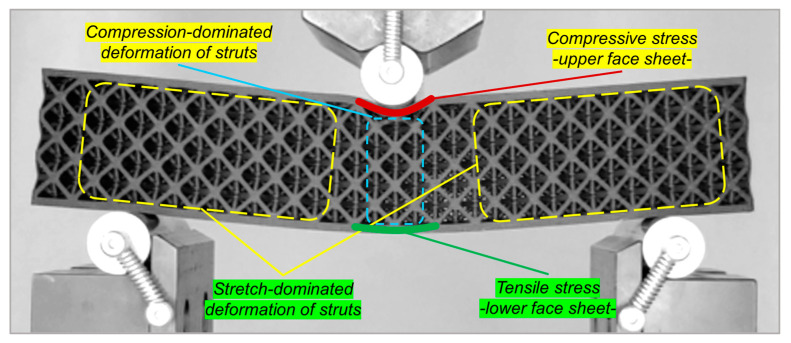
Different deformation types occurred in different regions of the octet lattice beam.

In this section, the deformation behavior of beams composed of lattice structures under a 3-point bending test is investigated. After a comprehensive analysis of the different stress types in lattice beams and the resultant deformations, the progressive deformation processes of both cube and octet lattice structures are illustrated alongside the appropriate spots on the force–displacement curves of the specimens. Figure 7 illustrates that there are fundamentally two distinct types of deformations present in the components of the lattice beam. The deformation can be categorized into two types: that which occurs in the upper and lower surface layers of the beam and that which takes place in the struts of the lattice. The deformations can be categorized into two types based on the kind of stress that induces the deformation. The deformation happening in the upper surface layer of the beam takes place under compressive stress (represented by the dark red section in the figure), whereas the deformation in the lower surface layer occurs under tensile stress (indicated by the green section in the figure). In the lattice struts, a deformation that is primarily influenced by compression takes place in the region where the indenter part makes contact (the blue hyphenated area), whereas a deformation that is mainly influenced by stretching occurs in the area between the indenter and the support parts (the yellow hyphenated areas in the figure). In this area, as the upper and lower layers shift their positions, the lattice struts experience simultaneous tensile and shear stresses. Therefore, the condition of the struts in the stretch-dominated area is a factor that directly influences the structural integrity of the lattice beam. Indeed, fractures and subsequent total failure took place in the areas of the vertical struts within the cube lattice that were in contact with the upper and lower surface layers of the beam. Conversely, it is recognized that the angled orientation of the struts within the octet lattice results in a more effective deformation response in the stretch-dominated area. Indeed, the absence of any cracks or ruptures in these lattice separations reinforces this situation.

**Figure 8:**
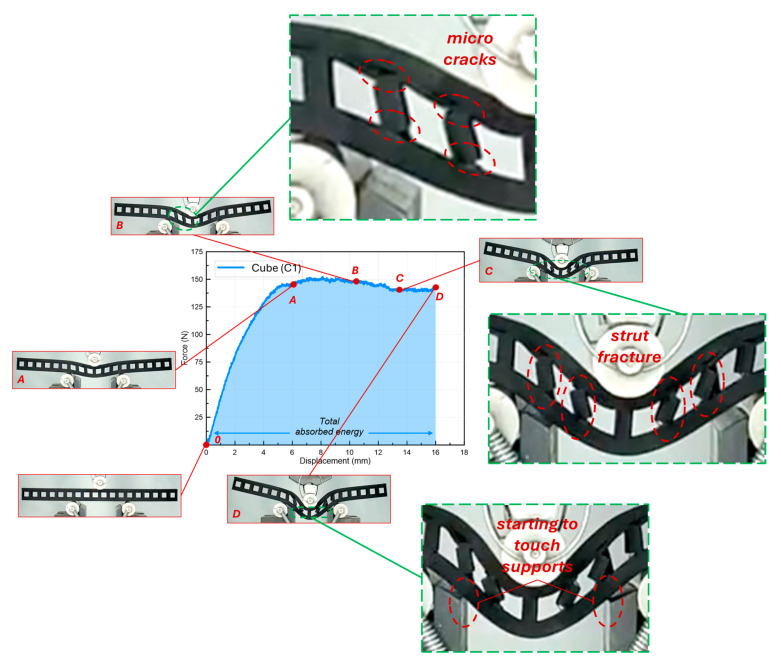
Force–displacement curve and progressive deformation stages of monolayer cube lattice beam.

Figure 8 shows the deformation stages of the cube lattice beam during the bending test. Accordingly, the indenter, which moves downward after the start of the experiment, tries to create a bending in the part of the lattice beam between the supports. Since the indenter contacts the beam at the beginning of the experiment, the force value increases immediately after the start of the experiment. At first glance, it can be seen that the force curve has a stable deformation behavior (plateau-like). The force increases gradually until the region is indicated by the letter A in the graph. At this time, it is seen that the part of the specimen in contact with the indenter deflects downwards (Figure 8). When the point indicated by the letter B in the graph is reached, it is seen that micro-cracks start to form in the struts located on the left and right sides of the indenter. Indeed, it can be said that the micro-cracks at the point where the lattice struts contact the top surface of the beam cause an almost imperceptible drop in the force curve. During the process, a decrease of almost 10 N in the force curve is noted at point C, which is attained after a relatively minor displacement value. The reason for this drop is plainly caused by the separation from the surface of the beam that can be seen at the struts, as can be seen from the detailed deformation picture that is shown in the graph. This deformation resulted in the beam’s diminished load-bearing capacity for a lower level. The cracks in the struts were located in the stretch-dominated zone, as previously stated. Consequently, the vertical struts could no longer withstand the tensile stress, resulting in tiny cracks at point C (Figure 8). Also, it can be said that the struts in the lattice beam have almost completely lost their load-carrying capability since struts do not contribute at all to the force transmission between the upper and lower surfaces of the beam. It can be said that this deformation behavior in the cube beam structure is based on the compressive stress that occurs in the region where the indenter part is in contact with the support parts, but this compressive stress turns into tensile stress as we move towards the support parts. At point D, the test was terminated since there was a minor gap between the lower face of the beam and the fixed supports (as can be seen from the deformation image in the graph).

**Figure 9:**
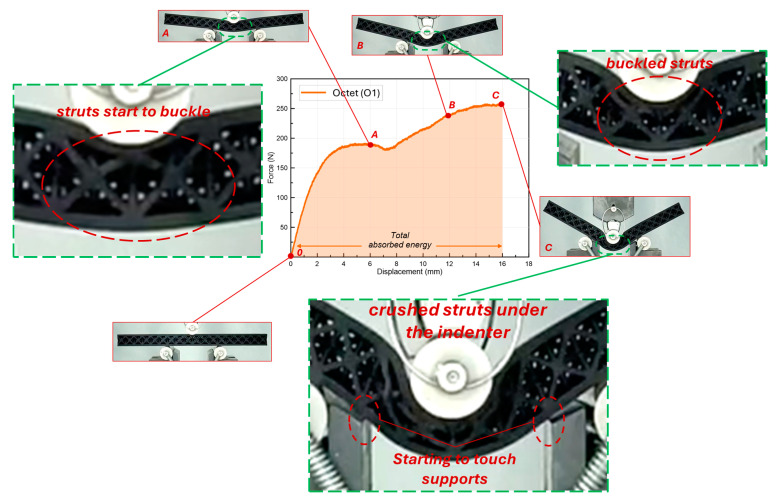
Force–displacement curve and progressive deformation stages of monolayer octet lattice beam.

Unlike the cube lattice beam, which has only vertical struts, the struts in the octet structure are positioned at an angle of 45°. Figure 9 shows the force–displacement curve of the monolayer octet lattice beam obtained from the 3-point bending test together with the deformation stages. It can be seen that the force value increases gradually from the beginning of the experiment (similar to the cube beam) and reaches point A. At this point, in the area where the indenter part contacts the beam, a local crushing of the beam under compressive stress has occurred, resulting in a decrease in the force curve. As a matter of fact, the local crushing that is brought about by the indenter part becomes more noticeable when point B is reached. The vertical struts in the cube beam, on the other hand, displayed a higher strength at the point of contact with the indenter. As a result, these specimens did not exhibit local crushing in the same way that the octet beams did (Figure 8). Conversely, the octet beam did not demonstrate the same separation or fracture as the cube beam. In this instance, it is clear that the angled octet struts have superior deformation resistance in the beam segment between the indenter and the fixed supports, where tensile stress (stretch-dominated deformation area) occurs. A slight increase can be observed after point B till point C, which is located at 16 mm of the displacement value. The test was completed at this stage as well (for the same reason as the cube beam).

### 3.2. Monolayer Beams: Single Column (ML-SC) and Double Column (ML-DC)

**Figure 10:**
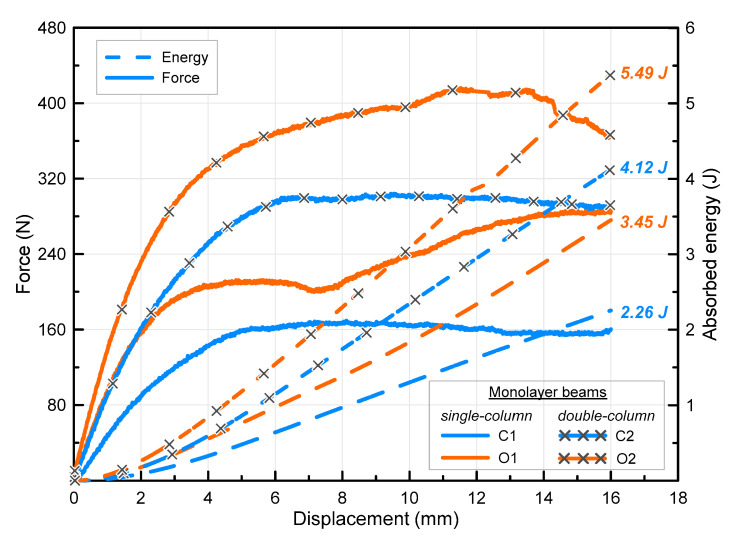
Force–displacement curves of monolayer single-column cube (C1) and octet (O1) lattice beams.

The force–displacement and energy–displacement curves of monolayer beams consisting of single- and double-column cube and octet lattices are presented together in Figure 10. The cube and octet lattice topologies were colored as dodger blue and orange for both single- and double-column beams, respectively. While all the specimens for each specimen configuration have the same line type for the force and energy, x symbols were used to represent the double-column beams. It is seen that the force curves have different characteristics for different topologies. The cubes have an almost stable force curve, while there is a gradual increase for octets. The curve of the octet beams, which is colored orange, appears to be displaced upwards compared to the curve of the cube beam counterparts. This is important because it shows that there is a significant difference in the stiffness values of the beams. It can be said that the octet beams have a higher stiffness (which can be identified as the ratio of force to displacement in the linear region from the beginning of the test). The stiffness ratios are equal to 40 N/mm and 90 N/mm for C1 and O1 lattice beams, respectively. It is seen that the octet lattice has more than double the stiffness compared to that of the cube lattice. This difference becomes more significant when one considers that the cube lattice has about 2.5 times the strut diameter of the octet. Therefore, it can be said that vertical struts, where buckling behavior is more dominant under axial load, exhibit lower performance than angled struts [16]. The C2 specimen has almost the same stiffness value as the O1 specimen, as can be seen from the figure. The stiffness ratio was reached at around 160 N/mm for the O2 beam, which is four times higher than the C1 beams. Considering the final displacement value of 16 mm, the beams absorbed 2.26 J and 3.45 J for C1 and O1, respectively. This shows that the single-column octet lattice beam absorbed 53% more energy compared to the cube one. The absorbed energy values for double-column cube and octet beams are equal to 4.12 J and 5.49 J, respectively. It is understood that the difference between the absorbed energy values decreases to 33% by using two columns side by side. In single-column cube beams, the cross-sections of the vertical struts are semicircular, whereas, in beams with two columns side by side, a full circular beam is formed in the center. This can be considered as the most obvious reason for the decrease in the difference between the absorbed energy values (from 53% to 33%) in cube and octet beams with the increasing number of columns. As a matter of fact, in octet lattices, since most of the angled separations in the lattice are in the form of a full circular cross-section, the use of two columns in these beams did not increase both the absorbed energy and stiffness ratios as much as their cube lattice counterparts. When considering the cube lattice has almost 2.5 times thicker struts (in diameter), octet struts exhibited higher performance in terms of energy absorption. Thus, the strut orientation becomes more dominant in this case.

### 3.3. Triple-Layer Double Column (TL-DC)

**Figure 11:**
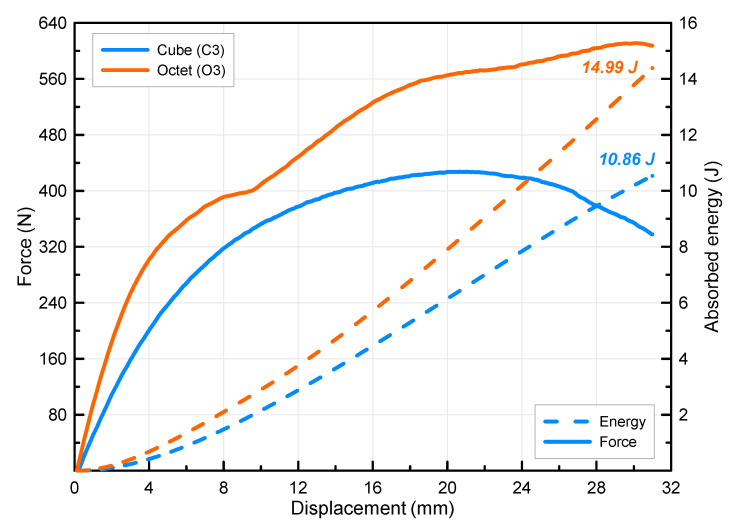
Force–displacement curves of triple-layer double-column cube (C3) and octet (O3) lattice beams.

In order to examine the effect of the number of layers on the lattice beams after seeing the effect of the side-by-side addition, 3-point bending tests of two-column and three-layer beams were carried out, and the data obtained as a result of the experiment were given together in Figure 11. As the number of lattices in the cross-sectional area increases, the curves become more stable, and no sudden drops are observed. Although there are no sudden decreases in the force curve of specimen C3 compared to the other specimen combinations (C1 and C2 specimens), the difference between the O3 and C3 curves has become much more obvious. It is also seen from the figure that the displacement values of the specimens corresponding to the maximum force value are also different from the other specimen combinations (30.5 mm for octet and 21.1 mm for cube). It is seen that the force values of the specimens are more than doubled in the cube specimen and approximately tripled in the octet specimen. It is understood that the absorbed energies of the specimens are quite different. The absorbed energy, which was 8.85 J in the cube specimen, increased around 70% more in the octet specimen and reached 14.99 J. The fact that the energy differences in the other specimen combinations are at low levels shows that the octet lattice is more dominant in terms of absorbed energy in the multi-layer beam design.

### 3.4. Sandwich Lattice Clusters

**Figure 12:**
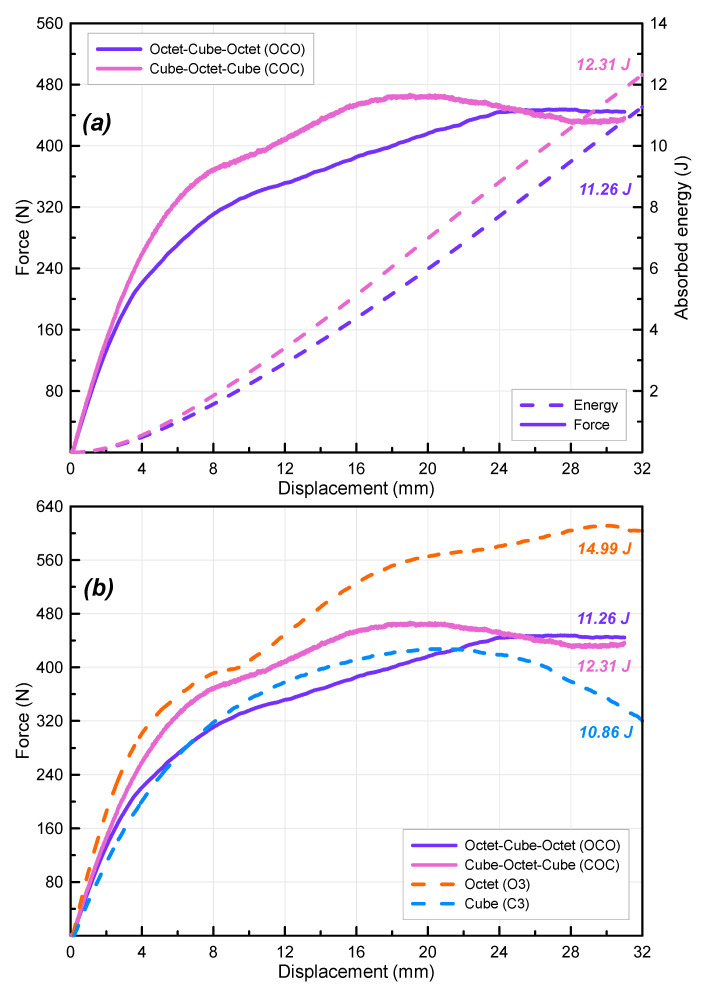
Force–displacement curves of triple-layer double-column (**a**) sandwich (cube-octet-cube (COC) and octet-cube-octet (OOC)) lattice beams and (**b**) comparison of uniform and sandwich beams.

For a better understanding of the effect of lattice type on the bending behavior of lattice beams, some triple-layer specimens were printed together using octet and cube lattices (Figure 12). The force–displacement curves of the COC and OCO specimens are given in Figure 12a. As can be seen from the graph, the use of different lattices together produced quite different results compared to uniform lattice structures. First of all, the change in the force curves of the specimens shows that the use of different lattices in beams directly affects the stiffness. The stiffness of the C3 beam reached a much higher level with octet lattice reinforcement (COC specimen). Although there is a small change in the displacement value reaching the maximum force, the decrease in the C3 force curve does not occur in the COC beam, indicating the contribution of the octet lattice to the load-carrying ability of the cube lattice. On the other hand, the force curve of the OCO beam using a cube lattice is quite different from that of the O3 beam. There is a decrease in the stiffness of the O3 specimen with the use of the cube lattice. Therefore, it can be said that the octet lattice has a significant contribution to both stiffness and load-carrying ability. There was also a significant difference in the energy values of the specimens. For example, the 12.31 J energy absorbed by the COC specimen is 13% higher than the uniform cube lattice specimen (C3), which shows the contribution of the octet lattice in the sandwich structure. Additionally, it is quite remarkable that the 14.99 J energy absorbed by the O3 beam specimen consisting of only an octet lattice decreased to 11.26 J in the OCO beam specimen. This shows that the uniform structure beam using an octet lattice is more efficient than the sandwich model. This can be explained by the fact that if different lattice types are used in different layers, the transmission of the applied force between the struts in different layers is less efficient than in the uniform case.

The crashworthiness parameters generated from the data obtained from the experimental study are given together in Table 4 below. Since the specimens were all produced at 30% relative density, the weights of beams with different topologies in the same specimen combinations are almost identical. Small deviations in the weights can be explained by the nature of the 3D printing method (such as residual filament). As mentioned previously, the weights of the beams were calculated for the beam between fixed supports. It can be deduced from the table that the highest energy is absorbed in the octet lattice type in each specimen group. With an increasing number of columns, the energy difference between the cube beam specimens becomes wider. This can be interpreted as a consequence of the fact that with the increasing number of columns in the cube lattices, a fully circular strut is formed in the center of the lattice. However, with the increasing number of layers, the difference between the absorbed energies reaches larger values in the octet lattice structure (the energy difference increases from 164% in the cube lattice to 173% in the octet lattice). The highest energy was obtained at 14.99 J in the O3 specimen. The energy decreased by about 25% to 11.26 J with the cube lattice substituted in the OCO specimen from the sandwich-structured beams. On the other hand, in the COC specimen, the absorbed energy increased by 13% with the use of an octet lattice. This change in absorbed energy is very important in terms of showing that the octet lattice is more efficient than the cube topology when used as a beam. In addition to these data, specific energy absorption and specific load-carrying capabilities were also evaluated. It can be seen that the SEA of the cube lattice beam reached 0.29 J/g from 0.33 J/g when using an octet lattice layer between the cube lattices (COC). Similarly, the SEA of the O3 specimen was decreased from 0.39 J/g to 0.30 J/g when using a cube middle layer. By considering the highest force values in the force curve of the beams, the SLC value was calculated by dividing this maximum force value by the weight of the beam. It is also as important as the SEA to show the load-carrying capability of the specimen per weight. It is seen that the highest SLC values were decreasing by increasing the column and layer numbers. This is important to show that the maximum force values were not as increased as the weight of the beam. Additionally, the highest values in SEA and SLC parameters were obtained in octet lattice beams in each specimen configuration.

**Figure 13:**
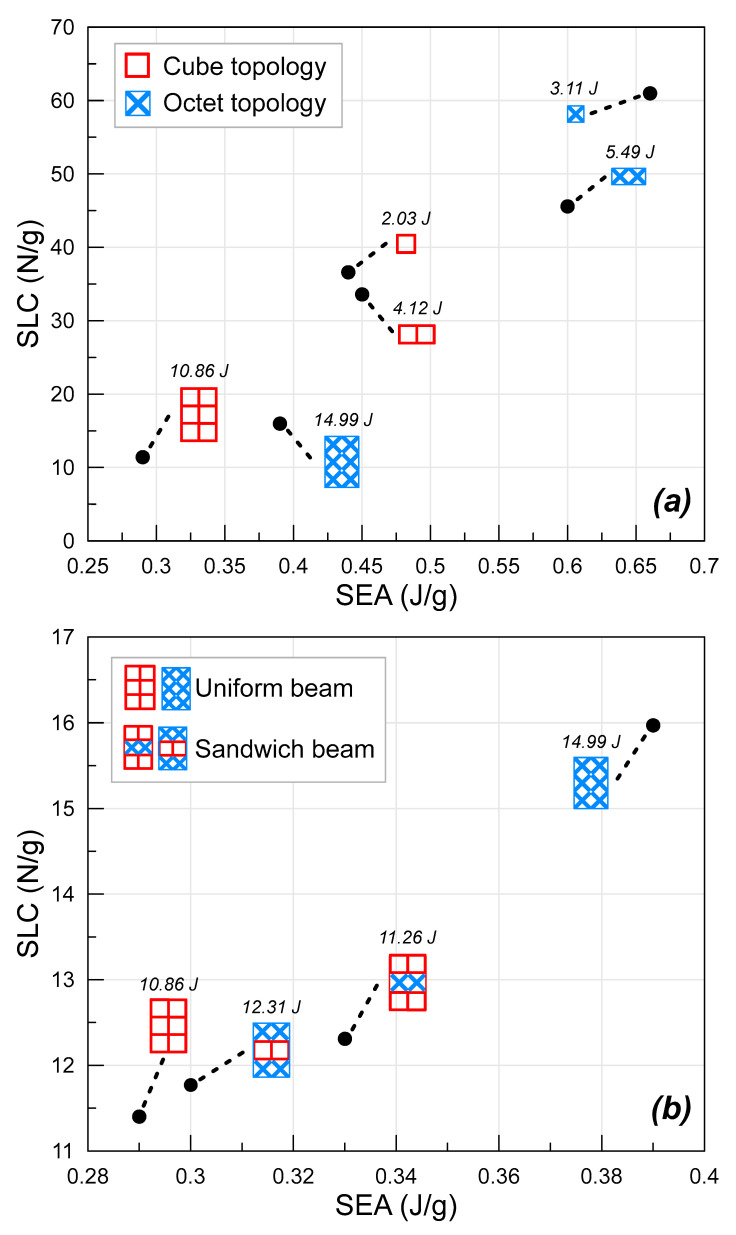
Specific load capacity and specific energy absorption comparison of (**a**) uniform lattice beams and (**b**) comparison of uniform and sandwich lattice beams.

SLC and SEA values of beams with uniform and sandwich lattice structures are given together in Figure 13. In general, it is clear that beams with octet lattices offer better results than beams with cube lattices in all specimen combinations (Figure 13a). The fact is that specimens C1 and C2 have almost the same SEA values, while C1 has a 9% higher SLC value compared to that of C2. This is very important, as it shows that an increasing column number is much more dominant on the absorbed energy than force when including the increase in the weight of the specimen. Similarly, the O1 specimen has 34% and 10% higher SLC and SEA, respectively, compared to the O2 specimen (Figure 13a). This shows that using the second column of the octet does not enhance the absorbed energy and the load-bearing capability, considering the increase in specimen weight. This is also important to show that the strut orientation has an important effect on the energy absorption capability. As can be seen from Figure 13b, the most efficient specimen combination in terms of the EA and SEA was the O3 beam with a uniform octet lattice. It can be said that this is followed by the OCO beam, where a cube lattice beam layer was placed in the center of two octet lattice beam layers. From Figure 13b, it is understood that the uniform octet beam (O3 specimen) has superior behavior in terms of the SEA and SLC, while the cube beam (C3 specimen) exhibits lower values for both of these parameters. As can be seen from the graph (Figure 13b), the SEA and SLC values of the specimens changed with different lattice-type substitutions to the uniform beams. For example, the OCO specimen obtained by substituting a cube lattice structure in the center of the octet beam has 27% lower SEA and 36% lower SLC values than the uniform O3 specimen. This can be explained by the fact that the vertical struts in the cube lattice have lower stability than the angled struts in the octet lattice. In contrast to the decrease in the uniform octet lattice, the SEA and SLC values increased by about 14% and 8%, respectively, with the substitution of the octet lattice to the uniform cube lattice.

Table 5 below shows the absorbed energy values of the beam specimens per column and per layer (C1 and O1 specimens are not considered layers since they are single columns). The per-column term was used to identify the beam consisting of a single column and single layers, such as C1 and O1, while the per-layer was used to show double columns and single layers as C2 and O2 specimens. To calculate the absorbed energy per column, the absorbed energy values were divided into two for monolayer double-column specimens and divided into six for triple-layer double-column beams. It is noteworthy that the pair of C2, C3 and O2, O3 beams have lower per-column absorbed energy values compared to the C1 and O1 beams. It is also interesting that the difference was becoming higher when using more columns. For instance, the absorbed energy per column was 2.06 J and 1.81 J for C2 and C3, respectively, while the absorbed energy value of the C1 beam was 2.26 J. A similar situation can be seen in octet beams as well. O2 and O3 beam specimens absorbed 2.70 J and 2.50 J, respectively, while the O1 specimen absorbed 3.45 J. When sandwich structures are considered, the absorbed energy per column decreased from 27% to 45% for the O3 and OCO specimens compared to the O1 specimen. This is important because it shows that the cube lattice substitution affects the absorbed energy. It is also seen that using an octet lattice with cube lattice beams could enhance the absorbed energy per column. By substituting the octet lattice between the cube lattices, the absorbed energy per column increased by around 10% compared to the uniform C3 beam specimen. Considering all these parameters, it can be said that using cube beams only side by side will give an efficient result for a possible beam design. In the case of octet beams, it is understood that both side-by-side and stacked use can be easily preferred. It can be said that this situation is directly related to the orientations of the octet and cube structures. As a matter of fact, the cube lattice, which has only vertical struts, exhibits a very low load-carrying performance in the bending test (especially in the tensile-dominated region). On the other hand, the octet lattice with 45-angled struts exhibits a more efficient load-carrying capability.

## 4. Conclusions

This study thoroughly investigated the bending characteristics of 3D-printed multi-topology lattice beams, with a specific emphasis on octet and cube lattice configurations. The experimental results demonstrate that the energy absorption, specific energy absorption (SEA), and specific load capacity (SLC) of the beams are greatly affected by the lattice topology, number of layers, and arrangement. In comparison to the cube lattice beams, the octet lattice beams consistently exhibited higher performance. For example, in the configuration of a triple-layer double column (TL-DC), the octet beam (O3) absorbed 14.99 J of energy, which is 38% more than the cube beam (C3), which absorbed 10.86 J. The O3 specimen exhibited a specific energy absorption (SEA) of 0.39 J/g, which was notably greater than the 0.29 J/g value of the C3 specimen. This suggests that the octet beam has a higher energy absorption capacity per unit mass. Furthermore, during the assessment of the monolayer double-column (ML-DC) beams, it was observed that the octet beam (O2) absorbed 5.49 J, which is approximately 33% greater than the 4.12 J absorbed by the cube beam (C2). The stability of the octet structures was further emphasized by the force–displacement curves, which showed a maximum load capacity of 611.3 N for the O3 specimen and 427.4 N for the C3 specimen. This indicates a 43% enhancement in load-carrying capability. Notably, the sandwich lattice arrangements, namely the octet-cube-octet (OCO) and cube-octet-cube (COC) beams, also exhibited intriguing outcomes. The OCO beam, comprising octet and cube structures, exhibited an absorption of 11.26 J, which was lower than the absorption of 14.99 J by the uniform octet lattice (O3) beam. These findings indicate that although the combination of various lattice designs can produce advantageous outcomes, the uniform octet structure remains the most efficient in terms of energy absorption and load-carrying capacity for the triple-layer beams. Additionally, two different deformation regions were identified for the beams as stretch dominated (occurred in the area between the indenter and supports) and compressive dominated (occurred in the area where the indenter touches to beam). It was observed that the vertical struts have a significant insufficiency in the stretch-dominated region due to the relative displacement change of the upper and lower layers of beams. Thus, the cube beams showed a lower load-bearing capacity compared to that of octet beams. However, the vertical struts play a better role in the compressive-dominated region.

### Proposed Future Considerations

- Material hybridization: In further research, the behavior of the investigated structures for various materials can be included in order to further improve the mechanical properties. The dynamic and impact loading response of the mentioned lattice structures can be considered.
- Environmental factors, including temperature and humidity, should be investigated to assess their impact on the performance of these lattice beams. Also, an investigation of the influence of manufacturing irregularities on the performance of lattice beams would be of great value.
- In order to further the understanding and use of 3D-printed lattice structures in engineering and other high-performance disciplines, these suggestions seek to expand upon the findings of this study.

## Figures and Tables

**Table 1:** Mechanical and physical properties of Onyx nylon-carbon filament [17].

Property	Value
Young’s Modulus, E	1400 MPa
$\mathrm{Yield}\;\mathrm{Strength},\;\sigma_{y}$	45 MPa
Poisson’s ratio, ν	0.4
$\mathrm{Density},\;\rho_{0}$	1200 kg/m^3^

**Table 2:** Three-dimensional printing parameters of lattice beams.

Parameters	Specifications
Nozzle temperature (°C)	273
Sliced layer thickness (mm)	0.1
Pattern	Solid
Density	100%

**Table 3:** The dimensions of different lattice beam configurations.

Beam Configurations			h (mm)	w (mm)	l (mm)
Monolayer single column	Uniform	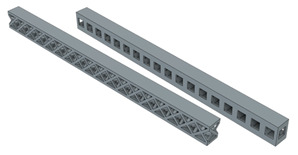	14	10	180
Monolayer double column	Uniform	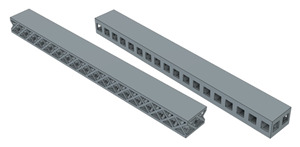	14	20	180
Triple-layer double column	Uniform	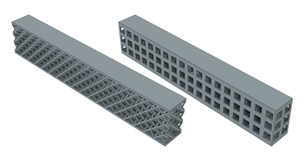	34	20	180
	Sandwich	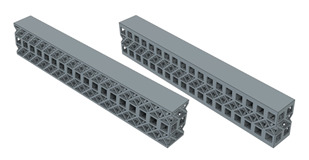	34	20	180

**Table 4:** Crashworthiness parameters of lattice beams with different topology.

Specimen Combination		Max Load Capacity LC, (N)	Weight (g)	Energy Absorption EA, (J)	Specific Load Capacity SLC, (N/g)	Specific Energy Absorption SEA, (J/g)
ML-SC beams	C1	169.4	4.63	2.03	36.59	0.44
		169.0	4.63	2.00	36.50	0.43
		170.0	4.63	2.05	36.72	0.44
	O1	286.7	4.70	3.11	61.00	0.66
		286.2	4.70	3.14	60.89	0.67
		287.0	4.70	3.09	61.06	0.66
ML-DC beams	C2	304.1	9.06	4.12	33.57	0.45
		304.0	9.06	4.10	33.55	0.45
		304.5	9.06	4.15	33.61	0.46
	O2	416.1	9.13	5.49	45.58	0.60
		415.8	9.13	5.45	45.54	0.60
		416.3	9.13	5.51	45.60	0.60
TL-DC beams	C3	427.4	37.49	10.86	11.40	0.29
		428.0	37.49	10.89	11.42	0.29
		426.8	37.49	10.80	11.38	0.29
	O3	611.3	38.27	14.99	15.97	0.39
		611.0	38.27	14.97	15.97	0.39
		611.5	38.27	14.98	15.98	0.39
Sandwich beams	OCO	447.7	38.03	11.26	11.77	0.30
		448.1	38.03	11.20	11.78	0.29
		447.4	38.03	11.29	11.76	0.30
	COC	466.9	37.80	12.31	12.35	0.33
		467.3	37.80	12.28	12.36	0.32
		466.5	37.80	12.36	12.34	0.33

**Table 5:** Total, per-layer, and per-column absorbed energy of lattice beams.

Specimen Combination		EA (J)	EA (Per Column) (J)	Differ. (%)	EA (Per Layer) (J)	Differ. (%)
SL-SC	C1	2.26	2.26	0	NA	NA
	O1	3.45	3.45	0	NA	NA
SL-DC	C2	4.12	2.06	−9	4.12	0
	O2	5.49	2.75	−20	5.49	0
TL-DC	C3	10.86	1.81	−19	3.62	−12
	O3	14.99	2.50	−27	5.00	−9
	OCO	11.26	1.88	−45	3.75	−32
	COC	12.31	2.05	−9	4.10	0

## Data Availability

The original contributions presented in the study are included in the article; further inquiries can be directed to the corresponding author.
